# Supplementary material for: Direct Observation of the Zigzag Edge States of a Supramolecular Diatomic Kagome Lattice
Source: Nano Lett. 2026 Sep 2;26(36):12146–52. doi: 10.1021/acs.nanolett.6c03195 (PMC13587768; doi:10.1021/acs.nanolett.6c03195)
Supplement: Supplementary file 1 [file nl6c03195_si_002.pdf]

Supporting Information for "Direct Observation of the Zigzag Edge States of a Supramolecular Diatomic Kagome Lattice"

Ryohei Nemoto,[†,‡] Xiangzhi Meng,[¶] Behzad Mortezapour,[¶] Alexander Weismann,[*,¶] Saya Nakano,[§] Masahisa Tsuchiizu,[§] Ryuichi Arafune,[†] Noriaki Takagi,[‖] Sho Nakamura,[⊥] Katsunori Wakabayashi,[†,⊥] Rie Suizu,[*,#,@,△] Richard Berndt,[¶] Takashi Uchihashi,[*,†,∇] and Kunio Awaga[††,@]

†*Research Center for Materials Nanoarchitectonics (MANA), National Institute for Materials Science, 1-1, Namiki, Tsukuba, Ibaraki 305-0044, Japan*

‡*Department of Physics, School of Science, Institute of Science Tokyo, 2-12-1, Ookayama, Meguro-ku, Tokyo 152-8551, Japan*

¶*Institut für Experimentelle und Angewandte Physik, Christian-Albrechts-Universität, 24098 Kiel, Germany*

§*Department of Physics, Nara Women's University, Kitauoyanishi-machi, Nara 630-8506, Japan*

‖*Graduate School of Human and Environmental Studies, Kyoto University, Kyoto 606-8501, Japan*

⊥*School of Science and Technology, Kwansei Gakuin University, Sanda, Hyogo 669-1330, Japan*

#*Synchrotron Light Application Center, Saga University, 1 Honjo, Saga, 840-8502, Japan*

@*Department of Chemistry and IRCCS, Nagoya University, Furo-cho, Chikusa-ku, Nagoya 464-8602, Japan*

△*Japan Science and Technology Agency (JST), PRESTO, 4-1-8 Honcho, Kawaguchi, Saitama, 332-0012, Japan*

∇*Graduate School of Science, Hokkaido University, Kita-10 Nishi-8, Kita-ku, Sapporo 060-0810, Japan*

††*National Institute of Technology (KOSEN), Toyota College, 2-1 Eiseicho, Toyota, Aichi 471-8525, Japan*

E-mail: weismann@physik.uni-kiel.de; rsuizu@cc.saga-u.ac.jp; UCHIHASHI.Takashi@nims.go.jp

Methods

Preparation and purification of Trip-Phz

Trip-Phz, molecule was prepared according to the procedure described in Ref.,[?] and was purified by sublimation at 550 K under a reduced pressure of 10^{-2} mbar.

Sample preparation and STM measurements

All samples were prepared in a UHV environment ($< 2.0 \times 10^{-10}$ mbar). A Pb(111) single crystal (MaTecK, 99.999%) was used as substrate. Atomically flat and clean Pb(111) surfaces were obtained through multiple cycles of Ar^+ sputtering (1.5 kV, 9 μA) and annealing (∼460 K). The Trip-Phz molecules were deposited on the Pb(111) surface through thermal sublimation from a home-built evaporator. The deposition rate was set at ∼0.3 ML min^{-1}. Depositing Trip-Phz for 60 seconds yielded approximately 0.3 ML of Trip-Phz, while the Pb(111) substrate was kept at room temperature.

All data were obtained with a low-temperature STM (4.6 K, Createc). The STM topographs were recorded in the constant-current mode. Both dI/dV spectroscopy and mapping were performed using the lock-in amplifier technique. The bias was modulated at 447 Hz with an amplitude of 10 mV, except for the data in Fig. S3 with an amplitude of 5 mV. The dI/dV images were acquired simultaneously with topographs using grid spectroscopy; dI/dV signal at a constant V were extracted from the grid data to create an image.

Tight-binding and DFT calculations

Tight-binding calculations were performed using fragment moieties, each of which was treated as a single orbital. The positions of the fragment centers are shown in Fig. 4(b). A periodic boundary condition was imposed in the y direction, while an open boundary condition was applied in the x direction. The size of the lattice was set to 6 and 128 sites in the x and y directions, respectively. The resulting band structure is independent of the choice of fragment

center positions. Additionally, the bulk band structure was calculated by imposing periodic boundary conditions in both the x and y directions. The band structure projected in the k_y space is displayed as a gray background in Fig. 4(a).

The energy levels and spatial distributions of LUMOs of Trip-Phz were calculated using the Gaussian 16 program package.[?] The energy difference between the LUMO and LUMO+1 was identified with $3t_0$, where t_0 is the intramolecular coupling. We also calculated the energy levels of an isolated pair of phenazine moieties. The atomic coordinates were taken from the results of DFT calculations described below. The energy difference was identified with $2t_1$, where t_1 is the intermolecular coupling. The values of $t_0 = -0.141$ eV and $t_1 = -0.096$ eV determined this way are consistent with our spectroscopic data; in Fig. 2(d), the two peaks corresponding to the LUMO and LUMO+1 states are separated by 0.3 eV, while they are broadened to 0.2 V. These values are close to $3|t_0| = 0.423$ eV and $2|t_1| = 0.192$ eV.

The geometry of Trip-Phz molecules adsorbed on the Pb(111) surface was determined by DFT calculations using the Vienna Ab initio Simulation Package (VASP)[?,?] with the projected augmented wave (PAW) potentials[?] and the generalized gradient approximation (GGA). The employed exchange-correlation functional was Perdew-Burke-Ernzerhof (PBE),[?] which incorporates nonlocal van der Waals interaction (rev-vdW-DF2).[?] The plane-wave basis set energy cutoff was set at 400 eV. Modeling Trip-Phz on Pb(111) involved using a supercell consisting of two Trip-Phz molecules on a three-layer Pb(111)-($\sqrt{31} \times \sqrt{31}$) slab, with a vacuum thickness of approximately 16 Å along the surface normal. To determine the equilibrium crystal structure, a conjugate-gradient relaxation of ionic positions was performed until the Hellmann-Feynman force on each atom was less than 0.02eV/Å, except for the bottom Pb layer. The atoms in the bottom layer were held at their ideal bulk positions during structure optimization. For self-consistent total energy calculations, the Brillouin zone was sampled using Monkhorst-Pack grids[?] with $9 \times 9 \times 1$ k-points.

Supporting Figures

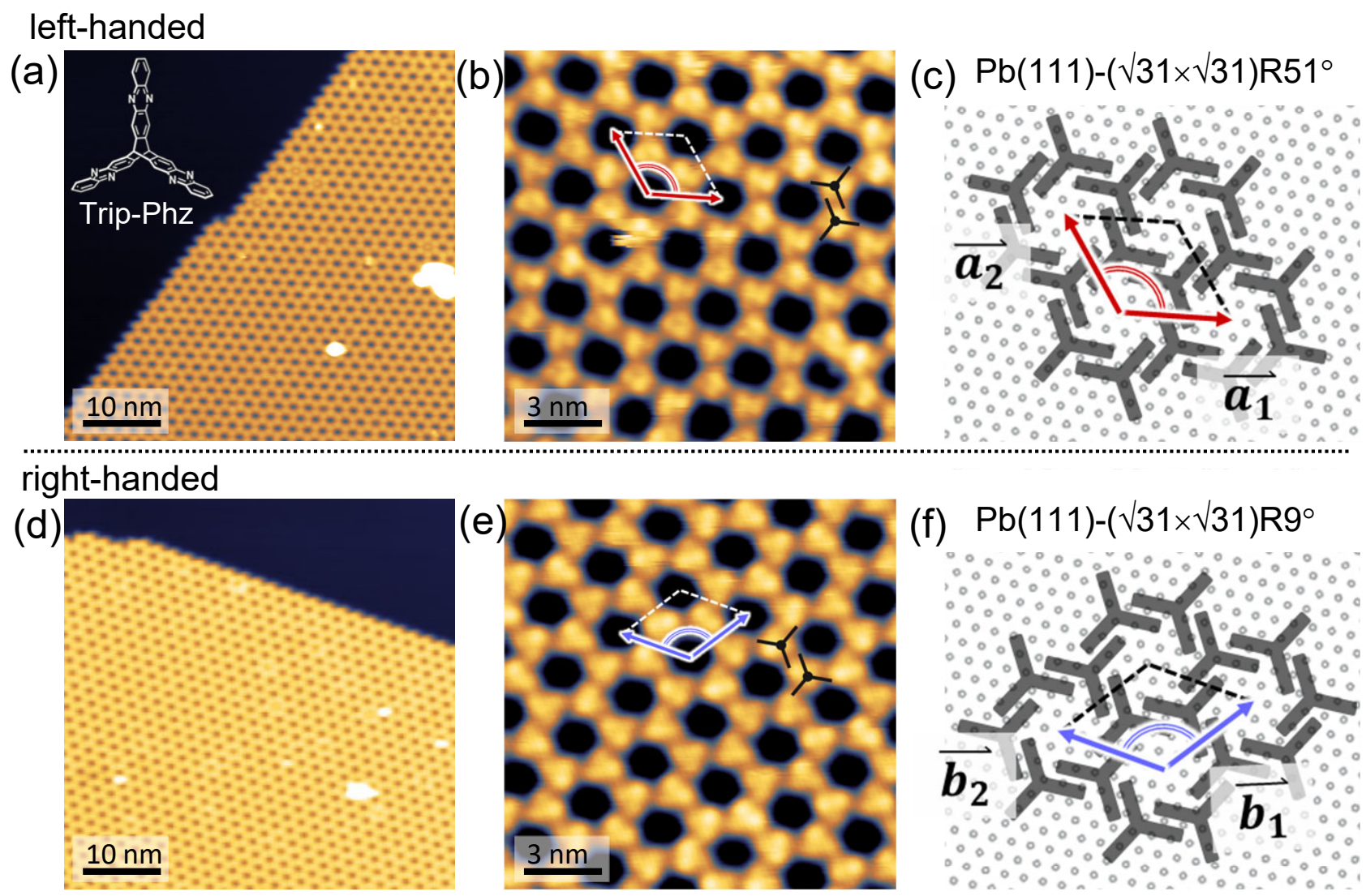

Figure S1: Chirality of self-assembled lattices of Trip-Phz molecules on Pb(111). (a-c) left-handed, (d-f) right-handed. (a, d) Large-area STM topographs recorded using $I = 10$ pA, (a) $V = 1.8$ V and (b) 1.5 V. (b, e) Detailed topographs overlaid with a unit cell (dashed rhombus) and lattice vectors. The molecular adsorption geometries are denoted by black Y-shaped lines. (c, f) Structural models illustrating the commensurate registry with the Pb(111) surface. While the unit cell dimensions are identical ($\sqrt{31} \times \sqrt{31}$), the angles with respect to the substrate lattice are (c) 51° and (f) 9°. The inset in (a) shows the chemical structure of Trip-Phz. The images shown in (a) and (b) are identical to those of Figs. 1(a) and 1(b).

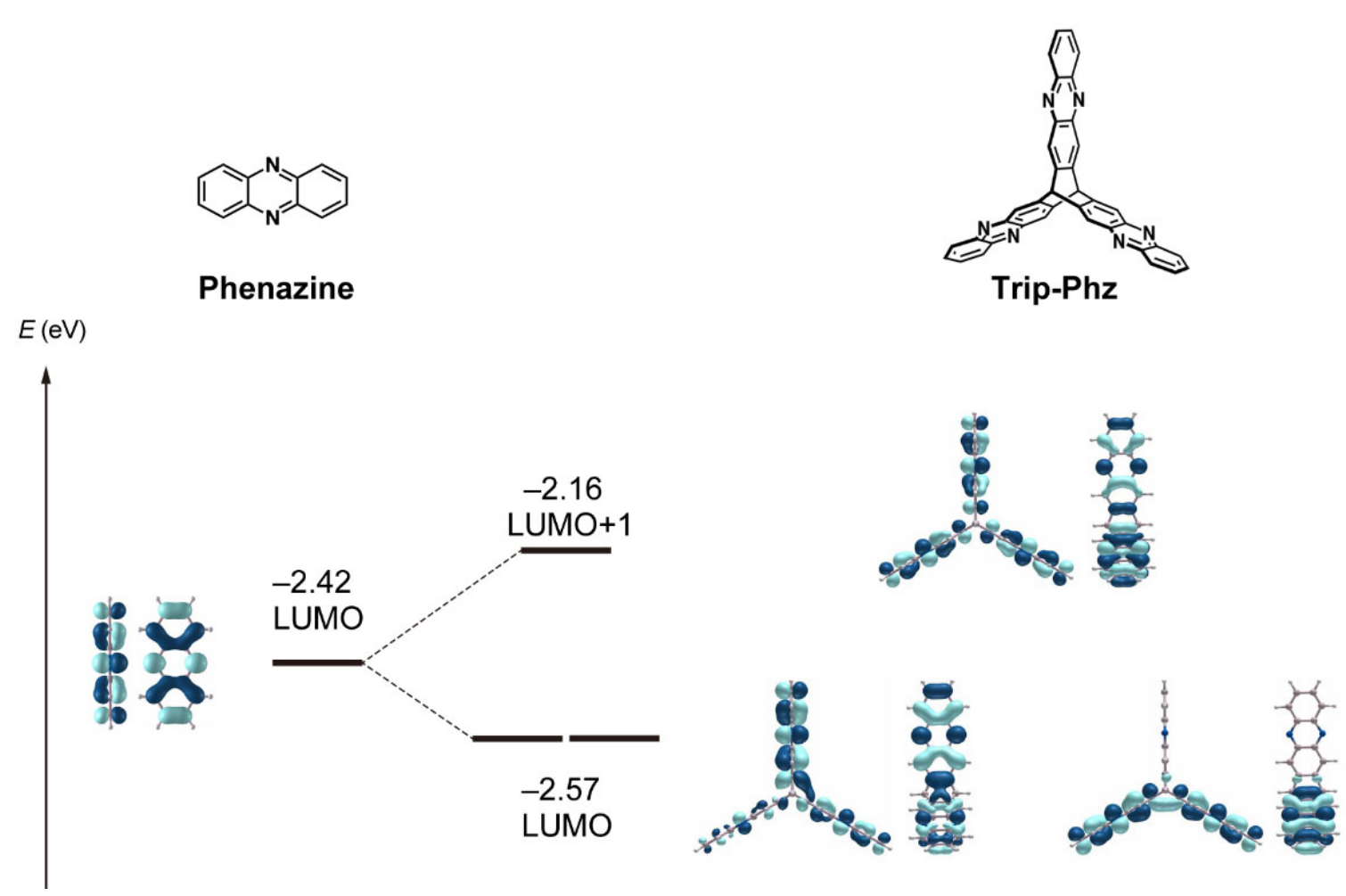

Figure S2: Energy levels and spatial distributions of LUMOs. (left) Phenazine moiety, (right) Trip-Phz molecule. Dark and light blue colors indicate the sign of wavefunction. The molecular orbital energies are shown in eV relative to the vacuum level.

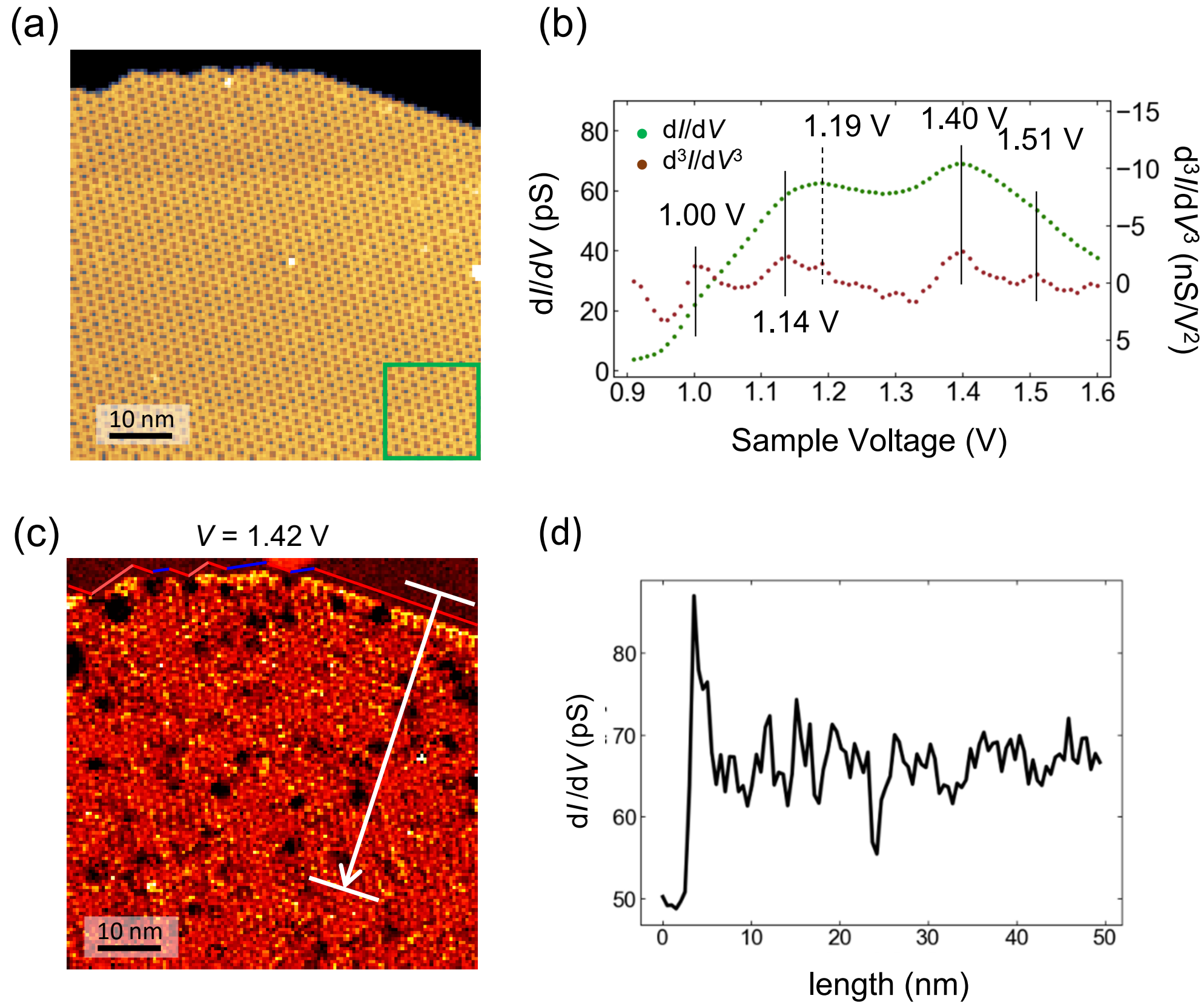

Figure S3: dI/dV spectra obtained in a bulk region of a Trip-Phz lattice. (a) STM topograph of Trip-Phz lattice recorded using $V = 1.6$ V, $I = 50$ pA. (b) dI/dV spectrum acquired within the green square in (a) (green dots). The positions of the peak and shoulders were determined from d^3I/dV^3 (brown dots), which was obtained by taking the second derivative of dI/dV with respect to V. (c) dI/dV image acquired at $V = 1.42$ V in the same area as in (a). (d) Line profile of dI/dV signal taken along the arrow line displayed in (c).

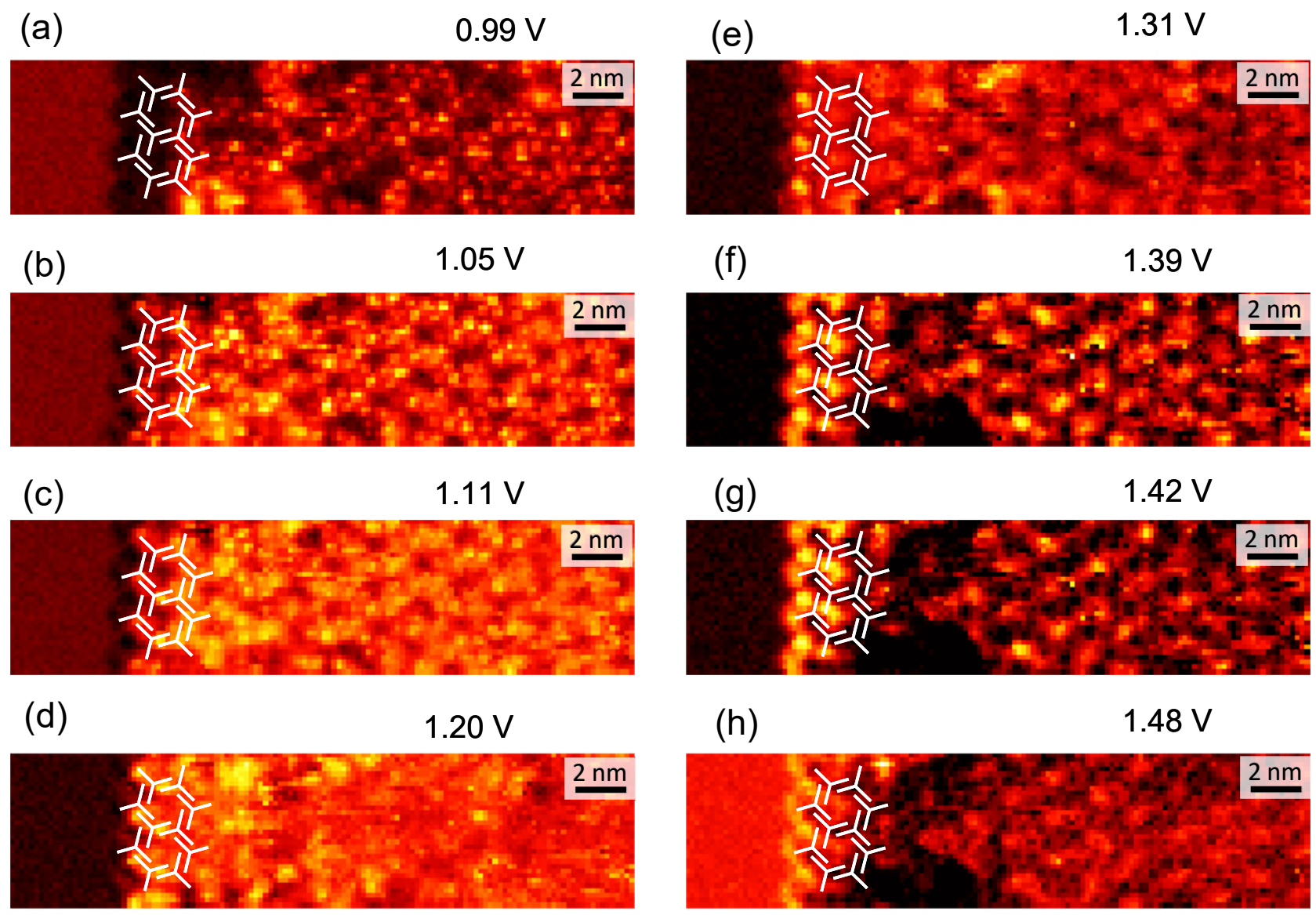

Figure S4: Bias voltage dependence of dI/dV images acquired over the same area as in Fig. 3(a). (a) $V = 0.99$ V, (b) $V = 1.05$ V, (c) $V = 1.11$ V, (d) $V = 1.20$ V, (e) $V = 1.31$ V, (f) $V = 1.39$ V, (g) $V = 1.42$ V, (h) $V = 1.48$ V. (g) is identical to Fig. 3(b).

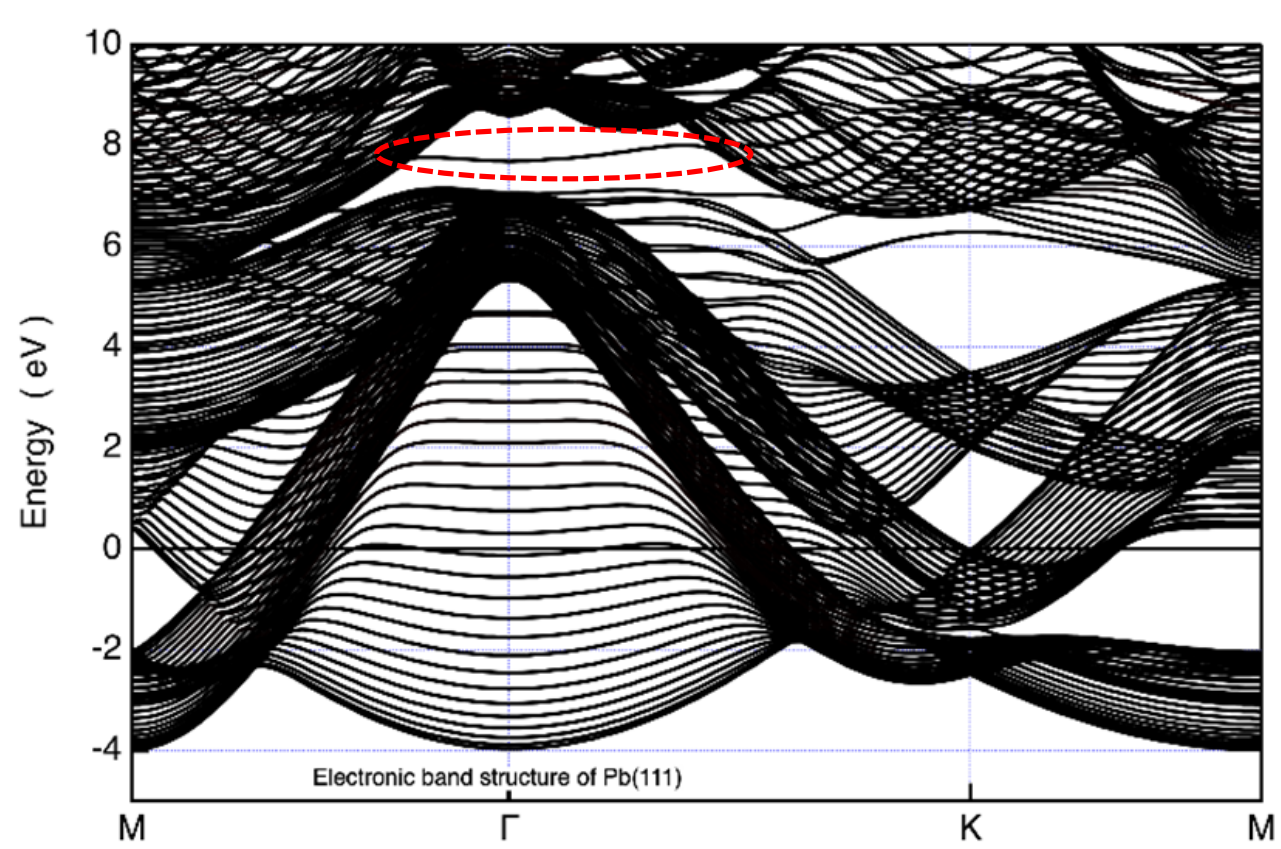

Figure S5: Electronic band structure of Pb(111). The Shockley surface states are located about 8 eV above the Fermi level (dashed red line). This prevents them from hybridizing with the molecular states located within 1-2 eV in this work.

Supporting Movie

MovieS1.mg4 shows the bias voltage dependence of dI/dV images acquired over the same area as in Fig. 3(a), including the data in Fig. S4. The bias voltage V ranges from 0.9 V to 1.6 V.

Supporting Note 1: Chiral symmetry of graphene

Within the framework of tight-binding approximation, the eigenstates of an electron in a periodic lattice can be written as $\Psi(\boldsymbol{R}) = u(\boldsymbol{k})e^{i\boldsymbol{k}\cdot\boldsymbol{R}}$, where $\boldsymbol{R}$ is the coordination of the atomic site, $\boldsymbol{k}$ is crystal momentum, and $u(\boldsymbol{k})$ is the amplitude of the wavefunction. The corresponding eigenenergy is $E(\boldsymbol{k})$. Since the honeycomb lattice of graphene has two independent atomic sites A and B within the unit cell, $E(\boldsymbol{k})$ is obtained by solving a Schrödinger equation of the following matrix form

$$H(\boldsymbol{k})\begin{pmatrix} u_A(\boldsymbol{k}) \\ u_B(\boldsymbol{k}) \end{pmatrix} = E(\boldsymbol{k})\begin{pmatrix} u_A(\boldsymbol{k}) \\ u_B(\boldsymbol{k}) \end{pmatrix}. \tag{1}$$

$H(\boldsymbol{k})$ is given by

$$H(\boldsymbol{k}) = \begin{pmatrix} 0 & h(\boldsymbol{k}) \\ h(\boldsymbol{k})^* & 0 \end{pmatrix} \tag{2}$$

$$h(\boldsymbol{k}) = -t\sum_{l=1}^{3} e^{i\boldsymbol{k}\cdot\boldsymbol{\tau}_l}, \tag{3}$$

where t is the transfer integral between the neighboring A and B sites, and $\boldsymbol{\tau}_l$ ($l = 1, 2, 3$) are the displacement vectors between them (see Fig. S6). $H(\boldsymbol{k})$ is referred to as a Bloch Hamiltonian.

The chiral symmetry of graphene can be expressed by an anticommutation relation of

the form

$$\{H, \Gamma\} = H\Gamma + \Gamma H = 0 \quad (4)$$

$$\Gamma = \begin{pmatrix} 1 & 0 \\ 0 & -1 \end{pmatrix}. \quad (5)$$

This symmetry of the Hamiltonian has important consequences. It leads to the formation of two independent, degenerate sets of Dirac cones at the K and K' points, which have opposite chiralities in terms of pseudospin rotation around them. It also results in the formation of a chiral-zero mode, whose energy is locked at $E = 0$. The zigzag edge state of graphene is an example of this. Thus, the chiral symmetry is considered a fundamental property of graphene.[?]

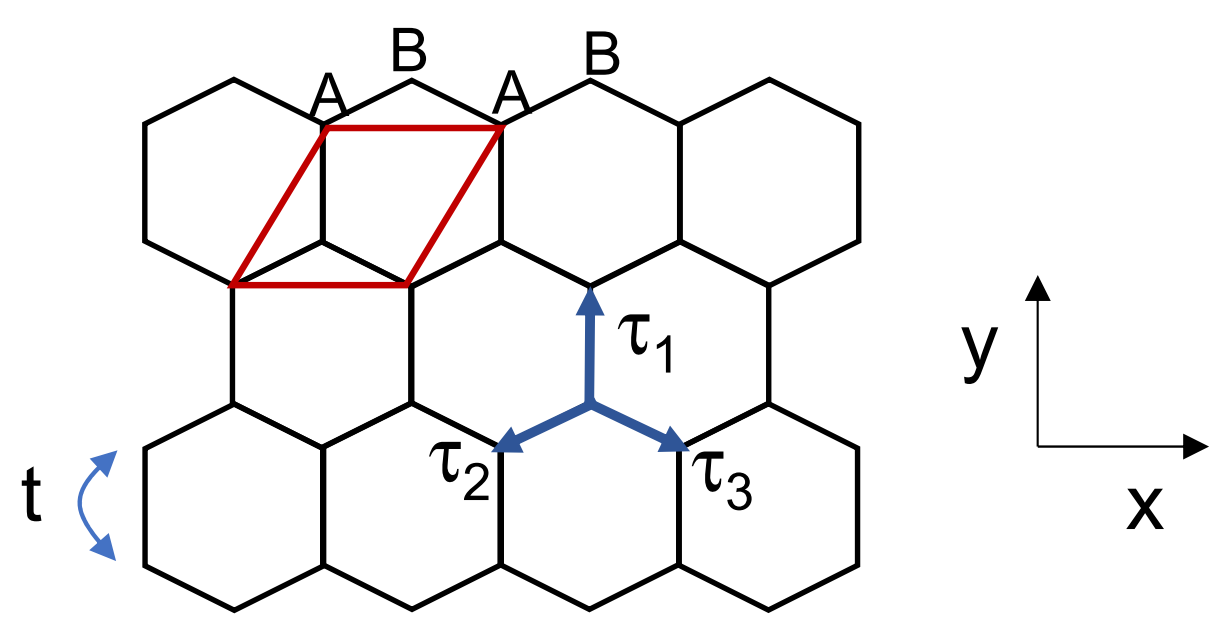

$\boldsymbol{k} = (k_x, k_y)$

Figure S6: Schematic model of the honeycomb lattice of graphene.

Supporting Note 2: Molecule-substrate interaction and the effect of spin-orbit interaction

Figure 2(d) shows that there exist shoulders in the spectrum acquired on the Pb(111) surface, which are located at energies close to those of the Trip-Phz spectral peaks ($V = 1.1, 1.4$ V). This suggests a possible enhancement of the molecule-substrate interaction. However, the

states around $1.1 - 1.4$ V observed on Pb(111) are not Shockley surface states, as DFT calculations reveals the absence of such surface-localized states in this energy range (see Fig. S5 in Supporting Information); the observed peaks originate only from bulk states. Furthermore, the LUMOs of the Trip-Phz molecule are protruding parallel to the Pb(111) surface as explained in the main text, severely limiting their interaction. Consequently, the spatial overlap and hybridization between the molecule and Pb(111) states at $1.1 - 1.4$ V should be negligibly small.

The above argument also indicates that spin-orbit interaction induced in the Trip-Phz molecules due to the heavy-atom Pb(111) substrate is also negligible, and that the observed edge states are not ascribed to this effect. It is well known that spin-orbit interaction opens an energy gap at the Dirac points and induces topological edge states within the gap.[?] However, since the resultant energy gap should also be small compared to temperature- and disorder-induced energy broadening, the emergence of topological edge states due to the spin-orbit coupling is very unlikely. By contrast, in the present case, the edge states are formed within an *intrinsic* gap between bulk bands due to the Zak phase quantization, which does not require the presence of spin-orbit coupling. Therefore, the observed edge states can safely be ascribed to those due to the Zak phase quantization.

Supporting Note 3: An intuitive picture of the Zak phase

The Zak phase is a Berry phase defined for a one-dimensional (1D) periodic system. Although the diatomic Kagome lattice is two-dimensional (2D), the crystal momentum parallel to the zigzag edge (k_y) remains a good quantum number. Therefore, for each fixed value of k_y, the two-dimensional system can be regarded as an effective 1D crystal extending along the x direction (Fig. S7(a)). The Zak phase is then calculated by integrating the Berry connection over the corresponding 1D Brillouin zone.

An intuitive interpretation of the Zak phase can be obtained from the concept of the Wannier center, which represents the center of the occupied electronic wave functions within a unit cell. Rather than describing the energy spectrum, the Zak phase characterizes the spatial distribution of the electronic wave functions in the bulk crystal.

For a crystal possessing inversion symmetry, the Wannier center is restricted to symmetry-related positions. Consequently, the Zak phase is quantized to either 0 or π (mod 2π). A Zak phase of 0 corresponds to a Wannier center located near the center of the unit cell, whereas a Zak phase of π corresponds to a Wannier center shifted toward the boundary between neighboring unit cells (Fig. S7(b)).

This simple picture naturally explains the origin of edge states. When a crystal is terminated to form an edge, a Wannier center located at the boundary between neighboring unit cells is effectively cut by the termination (Fig. S7(c)). As a result, the electronic structure near the boundary cannot be completely reconstructed from the bulk states alone, leading to an electronic state localized at the edge. This mechanism is closely related to the end states of the Su-Schrieffer-Heeger (SSH) model,[?] although the present system is a two-dimensional diatomic Kagome lattice.

For the present zigzag termination, the total Zak phase becomes π in the momentum range where the ES5 edge state is observed, whereas it is zero elsewhere. Thus, the Zak phase provides an intuitive criterion for identifying the momentum interval in which the zigzag edge state can appear. The excellent agreement between the calculated Zak phase and the edge-state dispersion demonstrates the bulk-edge correspondence in the present system.

It should be emphasized that the Zak phase does not predict the energy of an edge state. Instead, it specifies whether a given one-dimensional subsystem possesses the appropriate bulk topology to support an edge-localized state for the chosen termination.

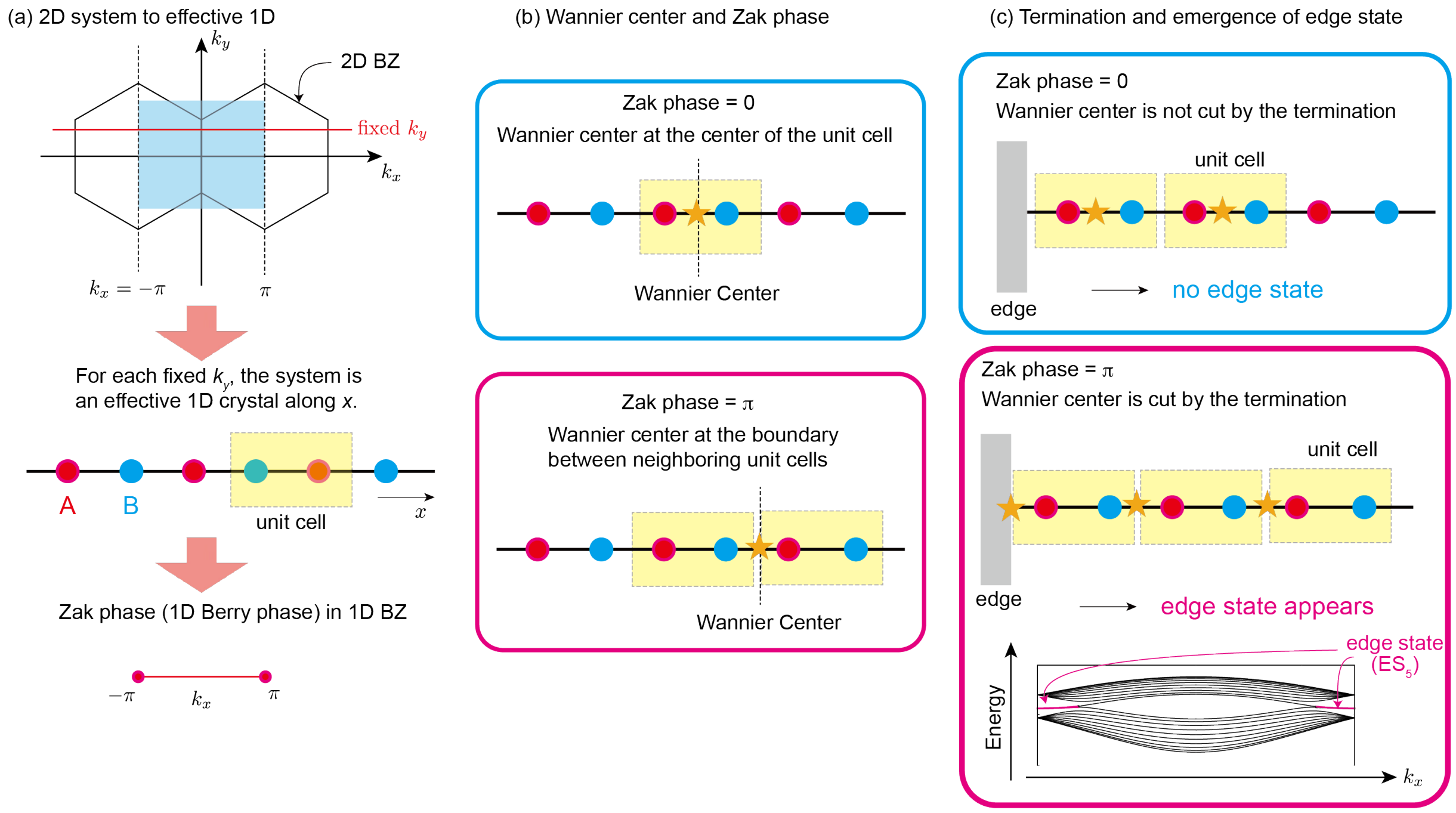

Figure S7: Intuitive picture of the Zak phase and the bulk-edge correspondence. (a) The two-dimensional diatomic Kagome lattice can be regarded as a family of effective one-dimensional systems parameterized by the conserved crystal momentum $k_y$parallel to the zigzag edge. For each fixed k_y, the Zak phase is calculated by integrating the Berry connection along the $k_x$direction across the corresponding one-dimensional Brillouin zone. (b) Schematic illustration of the Wannier center associated with the occupied electronic states. In the presence of inversion symmetry, the Zak phase is quantized to either 0 or π, corresponding to Wannier centers located at symmetry-related positions within the unit cell. (c) When the crystal is terminated to form a zigzag edge, a Wannier center located at the boundary between neighboring unit cells may be interrupted by the termination, giving rise to an edge-localized electronic state. This provides an intuitive picture of the bulk-edge correspondence discussed in the main text.
